# Supplementary material for: Evolution of the Growth Hormone Gene Duplication in Passerine Birds
Source: Genome Biol Evol. 2023 Feb 27;15(3):evad033. doi: 10.1093/gbe/evad033 (PMC10016047; doi:10.1093/gbe/evad033)
Supplement: evad033_Supplementary_Data [file evad033_supplementary_data.zip › Supplemental_Figures.pdf]

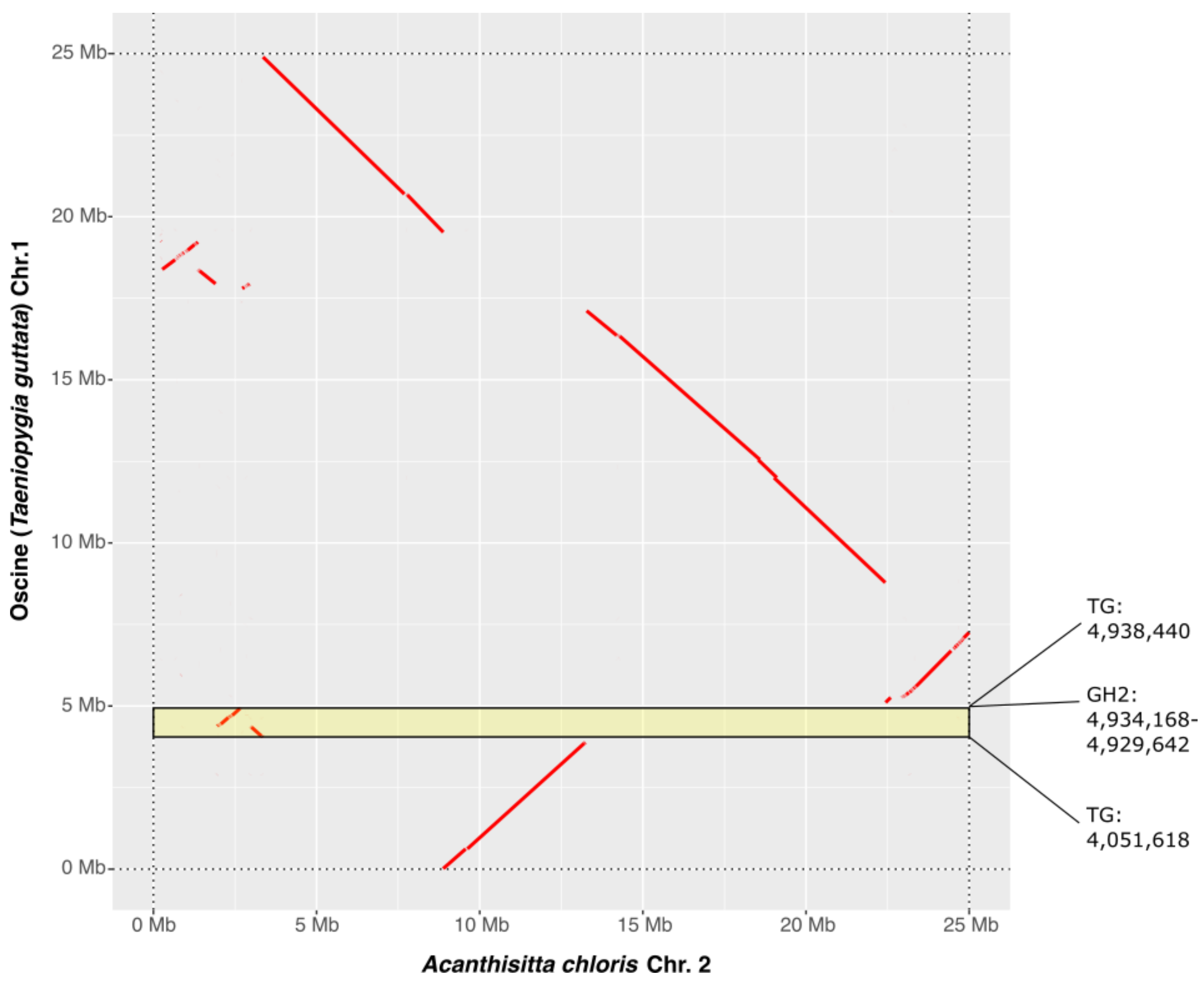

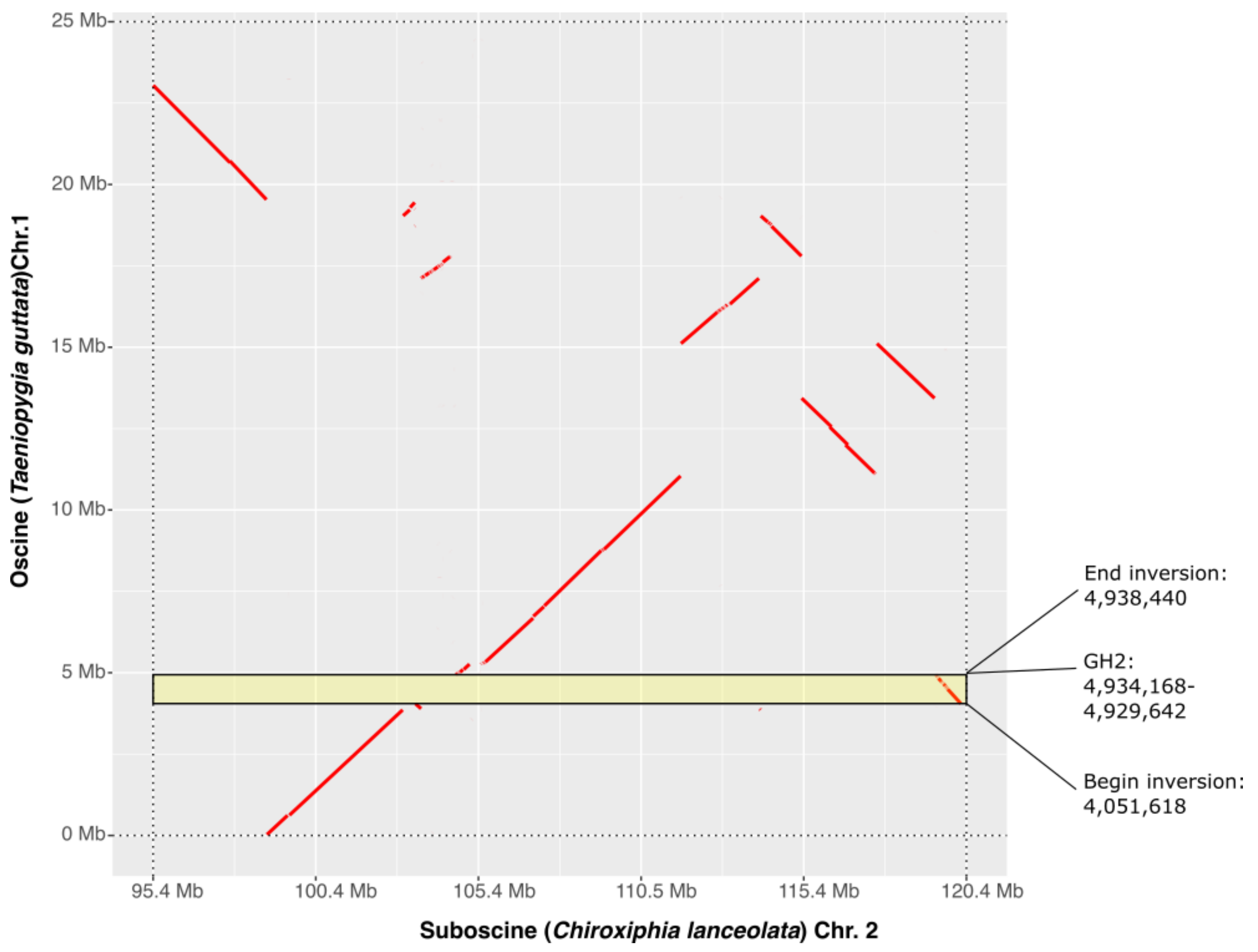

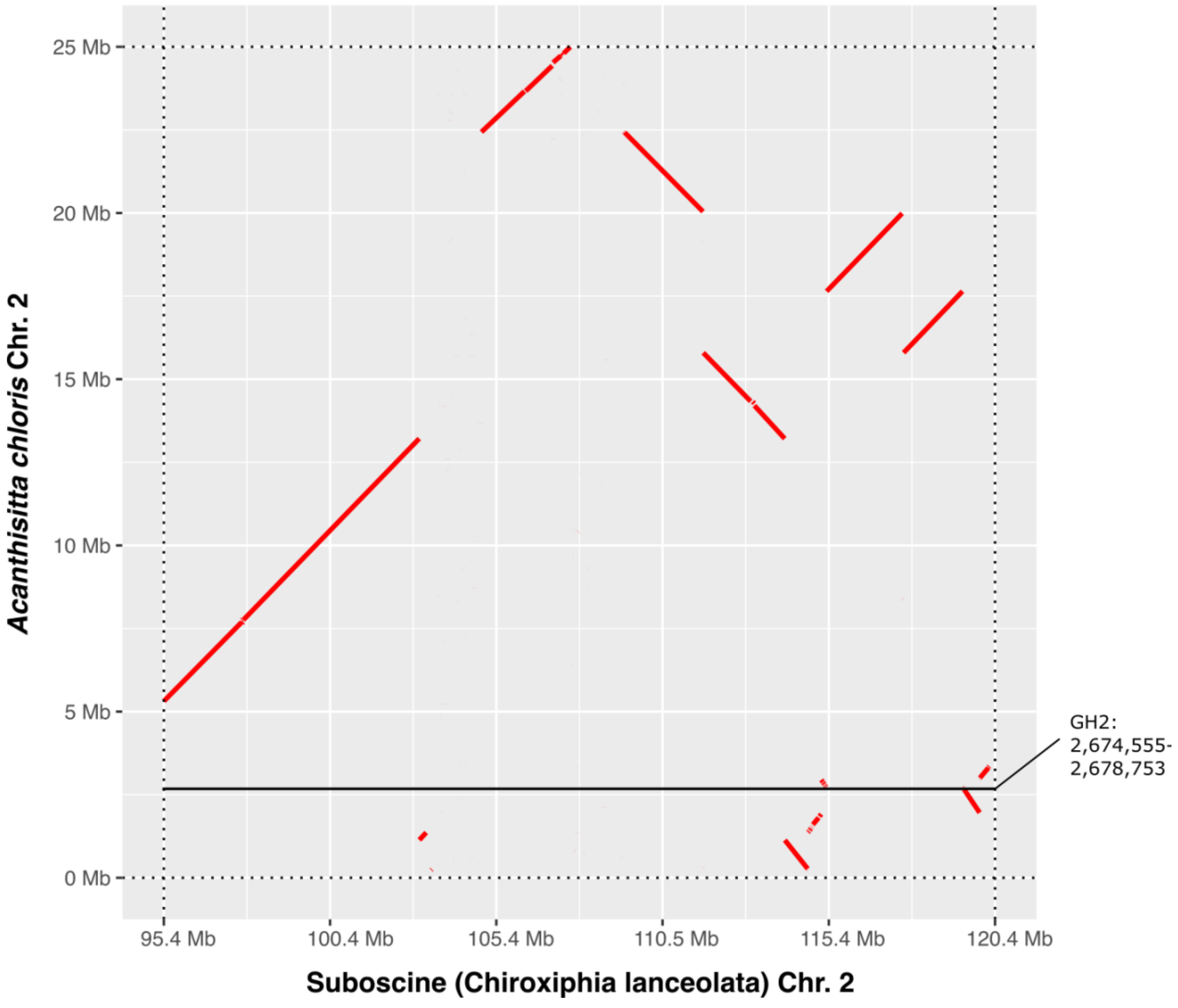

Supplemental Figure 1: Top left panel: *the first 25 megabases (Mb) and final 25 Mb of the oscine chromosome 1 and the homologous acanthisittine chromosome 2 are aligned and displayed in a dotplot. GH2 is on an inverted and displaced segment. The beginning and ending coordinates of the inversion in* Taeniopygia guttata *estimated in the alignment are labeled, with the coordinates for GH2 listed also. The inversion breakpoint is an estimated 4 kb from the GH2 translation start site.* ***Top right panel:*** *the first 25 Mb of the oscine chromosome 1 and the homologous suboscine chromosome 2 are aligned and displayed in a dotplot. GH2 is on a displaced segment. The highlighted box is for the same coordinates of* Taeniopygia guttata *chr. 1 in both top panels.* ***Bottom left panel:*** *the first 25 Mb of the acanthisittine chromosome 2 and the final 25 Mb of the suboscine chromosome 2 are aligned. The location of GH2 on* Acanthisitta chloris *chr. 2 is marked.*

Supplemental Figure 2: Tree Topologies used in tests of evolutionary rates and selection. **A.** The unconstrained gene tree topology obtained with RAxML using the growth hormone coding sequence alignment (Supplemental Data: GH CDS Alignment). **B.** The constrained topology obtained using the same CDS alignment as in A, but with the tree search constrained to trees compatible with an input topology. **C.** The input constraint topology used to obtain B. The input topology captures well-resolved and widely-accepted species relationships of birds based on prior studies, a fraction of which are cited below. RAxML settings used for A and B are in Methods: *Phylogenetic Tree Estimation*.

Literature:

Ericson PGP, Anderson CL, Britton T, Elzanowski A, Johansson US, Källersjö M, Ohlson JI, et al. 2006. Diversification of Neoaves: Integration of molecular sequence data and fossils. *Biol. Lett*. 2(4):543–547.

Gibson R, Baker A. 2012. Multiple gene sequences resolve phylogenetic relationships in the shorebird suborder Scolopaci (Aves: Charadriiformes). *Mol. Phylogenetics Evol.* 64(1):66–72.

Gonzalez J, Düttmann H, Wink M. 2009. Phylogenetic relationships based on two mitochondrial genes and hybridization patterns in Anatidae. *J. Zool.* 279(3):310–318.

Hackett SJ, Kimball RT, Reddy S, Bowie RCK, Braun EL, Braun MJ, Chojnowski JL, et al. 2008. A phylogenomic study of birds reveals their evolutionary history. *Science*. 320(5884):1763–1768.

Helbig AJ, Kocum A, Seibold I, Braun MJ. 2005. A multi-gene phylogeny of aquiline eagles (Aves: Accipitriformes) reveals extensive paraphyly at the genus level. *Mol. Phylogenetics Evol.* 35(1):147–164.

Irestedt M, Jønsson KA, Fjeldså J, Christidis L, Ericson PG. 2009. An unexpectedly long history of sexual selection in birds-of-paradise. *BMC Evol. Biol.* 9(235). https://doi.org/10.1186/1471-2148-9-235

Jarvis ED, Ye C, Liang S, Yan Z, Zepeda ML, Campos PF, Missael A, et al. 2014. A phylogeny of modern birds. *Science*, 346(6215), 1126–1138.

Johansson US, Ekman J, Bowie RCK, Halvarsson P, Ohlson JI, Price TD, Ericson PGP. 2013. A complete multilocus species phylogeny of the tits and chickadees (Aves: Paridae). *Mol. Phylogenetics Evol.* 69(3):852–860.

Ohlson JI, Fjeldså J, Ericson PGP. 2013. Molecular phylogeny of the manakins (Aves: Passeriformes: Pipridae), with a new classification and the description of a new genus. *Mol. Phylogenetics Evol.* 69(3):796–804.

Oliveros CH, Field DJ, Ksepka DT, Keith Barker F, Aleixo A, Andersen MJ, Alström et al. 2019. Earth history and the passerine superradiation. *Proc. Natl. Acad. Sci. U.S.A.* 116(16):7916–7925.

Prum RO, Berv JS, Dornburg A, Field DJ, Townsend JP, Lemmon EM, Lemmon AR. 2015. A comprehensive phylogeny of birds (Aves) using targeted next-generation DNA sequencing. *Nature*, 526(7574):569–573.

Reddy S, Kimball RT, Pandey A, Hosner PA, Braun MJ, Hackett, SJ, Han KL, et al. 2017. Why do phylogenomic data sets yield conflicting trees? Data type influences the avian tree of life more than taxon sampling. *Syst. Biol.* 66(4):857–879.

Sangster G, Alström P, Forsmark E, Olsson U. 2010. Multi-locus phylogenetic analysis of Old World chats and flycatchers reveals extensive paraphyly at family, subfamily and genus level (Aves: Muscicapidae). *Mol. Phylogenetics Evol.* 57(1):380–392.

Shen YY, Dai K, Cao X, Murphy RW, Shen XJ, Zhang YP. 2014. The updated phylogenies of the phasianidae based on combined data of nuclear and mitochondrial DNA. *PLoS ONE*, 9(4). https://doi.org/10.1371/journal.pone.0095786

Soares AER, Novak BJ, Haile J, Heupink TH, Fjeldså J, Gilbert MTP, Poinar H, et al. 2016. Complete mitochondrial genomes of living and extinct pigeons revise the timing of the columbiform radiation. *BMC Evol. Biol.* 16(1), 1–9.

Sun Z, Pan T, Hu C, Sun L, Ding H, Wang H, Zhang C, et al. 2017. Rapid and recent diversification patterns in Anseriformes birds: Inferred from molecular phylogeny and diversification analyses. *PLoS ONE*, 12(9). https://doi.org/10.1371/journal.pone.0184529

Valqui T. 2009. *Phylogeography of Nothoprocta tinamous and the phylogeny of the TINAMIDAE*. Dissertation. Lousiana State University.

A. Unconstrained Tree Topology

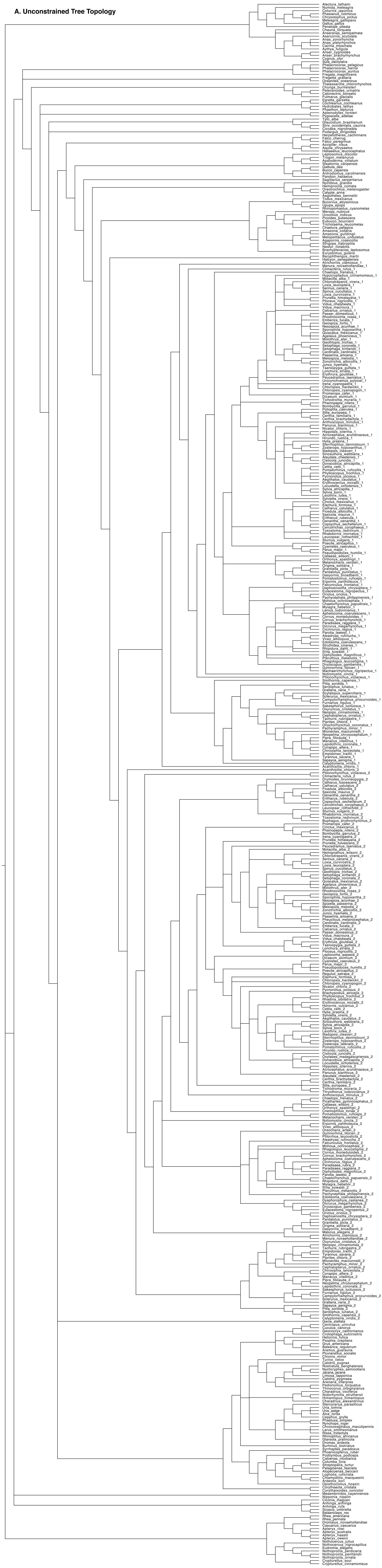

B. Constrained Tree Topology

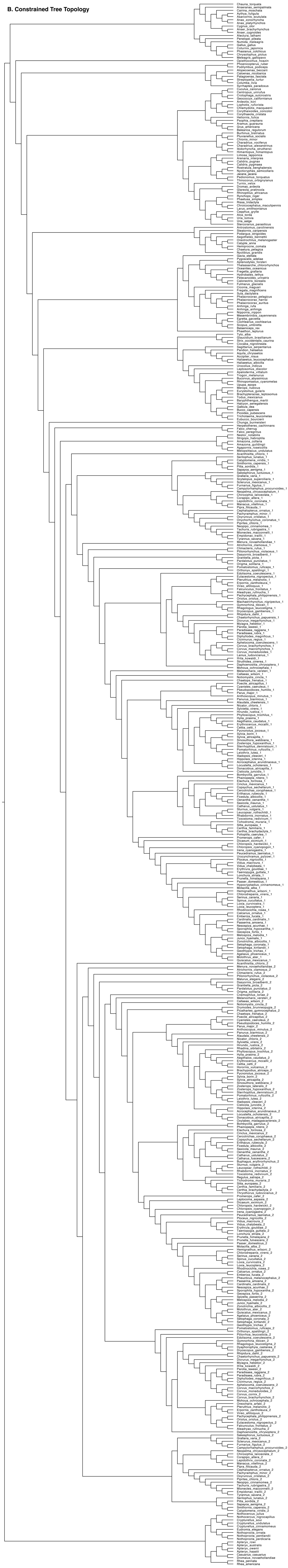

C. Constrained Tree Backbone

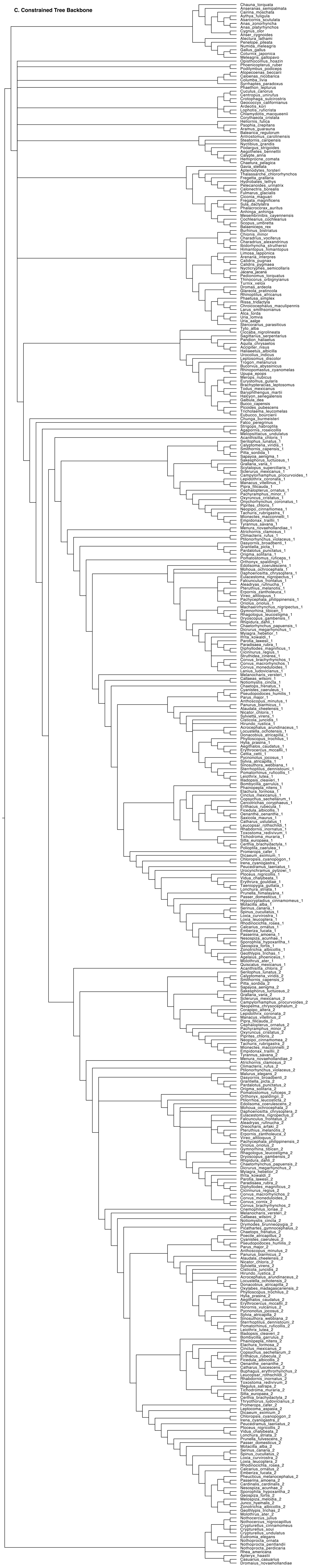

Supplemental Figure 3:
Phylogenetic Distribution of 15 bp Repeat in GH1 Promoter Region

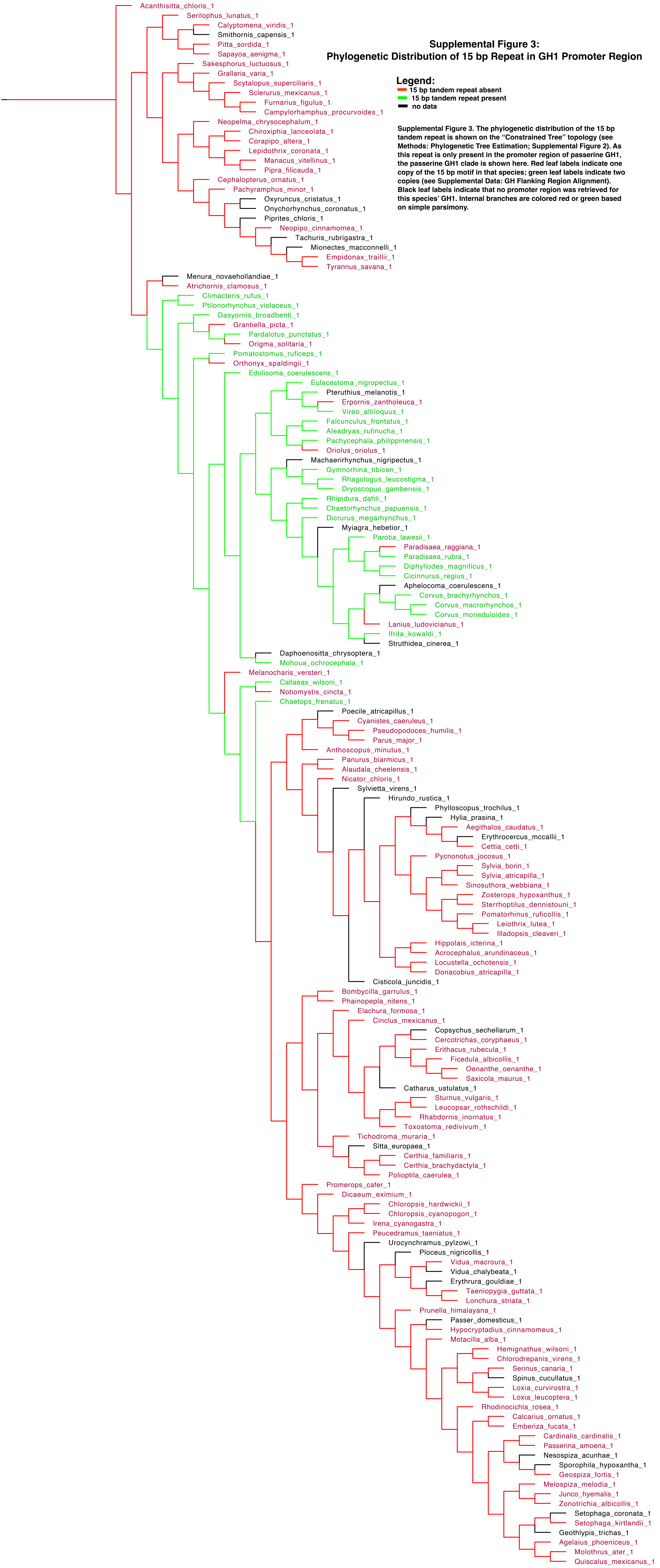

Supplemental Figure 3. The phylogenetic distribution of the 15 bp tandem repeat is shown on the "Constrained Tree" topology (see Methods: Phylogenetic Tree Estimation; Supplemental Figure 2). As this repeat is only present in the promoter region of passerine GH1, the passerine GH1 clade is shown here. Red leaf labels indicate one copy of the 15 bp motif in that species; green leaf labels indicate two copies (see Supplemental Data: GH Flanking Region Alignment). Black leaf labels indicate that no promoter region was retrieved for this species' GH1. Internal branches are colored red or green based on simple parsimony.
